# Comparing knowledge, attitudes and sexual practices of Female Commercial Sex Workers (FCSW) and the general female population in Brazil

**DOI:** 10.1186/1742-4690-9-S1-P108

**Published:** 2012-05-25

**Authors:** Célia Landmann Szwarcwald, Ana Roberta Pati, Pascom Paulo, Roberto Borges de Souza

**Affiliations:** 1 Fundação Oswaldo Cruz, Rio de Janeiro, Brazil

## Introduction

The aim of this study was to compare knowledge and risk behavioural practices among female commercial sex workers (FCSW) and the general female population in Brazil.

## Material and methods

Information on 2523 FCSW were collected in a RDS study carried out in 10 Brazilian cities in 2009. A method for estimating proportions and their variances was proposed, which takes into account the dependency structure of observations. Both the inverse of network size and the size of the city were considered in the estimation of RDS weights. The 2008 Behavioural Surveillance Study provided information of the general female population. The sample design of the BSS was a typical three stage-selection (census tracts, households and individuals), stratified by macro-region. In the statistical analysis, tests of proportions were performed taking into consideration each study sample design.

## Results

FCSW are less educated than the general female population aged 18 to 64 years. Knowledge of HIV transmission is always worse among FCSW, even after controlling for educational level. As to sexual practices, the onset of sexual activity is much earlier among FCSW and 38% reported sexual abuse at least once during lifetime. Although protected sexual practices showed a slightly more favourable scenario among FCSW, the proportion who would never fail to use a condom was very low (23%). Higher use of drugs among FCSW was another point of note. In relation to prevention measures, the coverage of gynaecological exam in the last three years was much higher in the general female population even though STI signs were more frequently reported among FSW. Similar proportions of HIV testing in the previous 12 months were found.

## Conclusions

The results showed that there are still shortcomings in preventive care among FCSW in Brazil. Measures specifically aimed at increasing knowledge of HIV transmission, coverage of gynaecological exam and annual HIV testing among FCSW could contribute to the reduction of STI transmission in the Brazilian population.

